# Laboratory infrared spectra and fragmentation chemistry of sulfur allotropes

**DOI:** 10.1038/s41467-024-50303-2

**Published:** 2024-07-15

**Authors:** Piero Ferrari, Giel Berden, Britta Redlich, Laurens B. F. M. Waters, Joost M. Bakker

**Affiliations:** 1 Radboud University, Institute for Molecules and Materials, FELIX Laboratory, Nijmegen, The Netherlands; 2 Department of Astrophysics, IMAPP, Radboud University, Nijmegen, The Netherlands

**Keywords:** Interstellar medium, Laboratory astrophysics, Chemical physics, Infrared spectroscopy

## Abstract

Sulfur is one of six life-essential elements, but its path from interstellar clouds to planets and their atmospheres is not well known. Astronomical observations in dense clouds have so far been able to trace only 1 percent of cosmic sulfur, in the form of gas phase molecules and volatile ices, with the missing sulfur expected to be locked in a currently unidentified form. The high sulfur abundances inferred in icy and rocky solar system bodies indicate that an efficient pathway must exist from volatile atomic sulfur in the diffuse interstellar medium to some form of refractory sulfur. One hypothesis is the formation of sulfur allotropes, particularly of the stable S_8_. However, experimental information about sulfur allotropes under astrochemically relevant conditions, needed to constrain their abundance, is lacking. Here, we report the laboratory far-infrared spectra of sulfur allotropes and examine their fragmentation pathways. The spectra, including that of cold, isolated S_8_ with three bands at 53.5, 41.3 and 21.1 µm, form a benchmark for computational modelling, which show a near-perfect match with the experiments. The experimental fragmentation pathways of sulfur allotropes, key information for astrochemical formation/destruction models, evidence a facile fragmentation of S_8_. These findings suggest the presence of sulfur allotropes distributions in interstellar space or in the atmosphere of planets, dependent on the environmental conditions.

## Abstract (web summary)

Sulfur allotropes are proposed to be an important sulfur reservoir in molecular clouds, but spectroscopic data to test this hypothesis are limited. Here the authors measure laboratory far-infrared spectra of cold isolated S_8_ molecules, which show a near-perfect match with calculations, and examine their fragmentation pathways.

## Introduction

Sulfur is the tenth most abundant element in the universe, with a cosmic [S/H] ratio of 1.4·10^−5^, which together with H, C, O, N and P, is regarded as one of the essential elements for life^1^. Moreover, sulfur is found in nature in a rich variety of allotropic forms, only surpassed in number by those of carbon^2^.

However, the pathway of sulfur from interstellar gas clouds to planets is a long-standing question^3–6^. While in low-density diffuse interstellar environments the observed atomic sulfur, expressed in its abundance ratio to atomic hydrogen, is close to the cosmic value^7,8^, in dense molecular clouds, star-forming regions and in planet forming disks that surround young stars, the gas phase sulfur concentration (measured as the sum of all atomic sulfur and sulfur atoms contained in gas phase molecules) is strongly depleted, by up to two orders of magnitude^9,10^. Volatile sulfur ice reservoirs such as OCS and SO_2_ can account for at most a few per cent of the missing sulfur^11^. In contrast, icy and rocky solar system bodies show abundant sulfur. Samples with a roughly solar [S/O] ratio were recovered by the Rosetta mission to the comet 67 P/Churyumov-Gerasimenko^12^, with about 80 per cent of sulfur in some refractory form. Refractory sulfur is also abundant in primitive meteorites of the CI class^13^, where it is found as sulfides and sulfates^14^. The sample return mission to the CI asteroid Ryugu^15^ has revealed similar high sulfur abundances. Recently, sulfur has for the first time been detected in the atmospheres of gas giant exoplanets^16,17^. These observations show that sulfur is efficiently converted from atomic to refractory reservoirs, even in icy objects like comets.

Sulfur allotropes have been proposed as an important sulfur reservoir in molecular clouds^18^, formed, for example, upon UV irradiation of H_2_S ices^19^, or electron irradiation of H_2_S and SO_2_ ices^20^. In the latter laboratory study, an apparent depletion of accountable sulfur budget was observed, attributed to the possible formation of sulfur allotropes. Indeed, S_2_, S_3_ and S_4_ were identified at trace abundances in 67 P/Churyumov-Gerasimenko^12^, measured with an instrument sensitive towards the small sulfur allotropes. Moreover, S_8_ was detected in Ryugu samples^21^. Sulfur allotropes may also play an important role in providing UV opacity in the Venusian atmosphere^22^, and in Venus-type exoplanets^23^. Density functional theory (DFT) calculations have suggested that among the S_*N*_ allotropes, octasulfur S_8_ is especially stable^24^, with astrochemical models highlighting its relevance in the dynamics of sulfur in the interstellar medium (ISM). Sulfur allotropes may therefore be the missing link between the diffuse interstellar medium and solar system parent bodies. Nevertheless, a lack of relevant spectroscopic and thermodynamic information on sulfur allotropes has prevented a proper investigation of this hypothesis as part of the solution of the sulfur puzzle. While sulfur allotropes have been the subject of active investigation^25–27^, spectroscopic information under astrochemically relevant conditions are scarce, with one example being the rotational spectra of S_3_ and S_4_^28^.

Here, we report the far-infrared spectrum of isolated neutral S_8_, under the cold and isolated conditions of a molecular beam. In addition, infrared spectra of the ions S_4_^+^ and S_4_^−^ are recorded in a room temperature ion trap. The experimental spectra of the investigated species show a remarkably good agreement with computational modelling, enabling us to predict lower abundance limits for their astronomical detection using the James Webb Space Telescope (JWST)^29^. These results can allow the targeted observational search of sulfur allotropes. The spectral information is complemented by information on fragmentation energetics and pathways providing necessary input for astrochemical modeling of the sulfur inventory in dense molecular clouds and star-forming regions.

## Results

### Far-infrared spectroscopy of the S_8_ allotrope

A cold and diluted molecular beam of neutral sulfur allotropes is generated from sulfur powder. A mass spectrum of the typically generated distribution (Supplementary Fig. 1) recorded after ionization with 118 nm laser light (10.5 eV/photon) shows allotropes from S_2_ to S_8_. The sulfur atom is not observed, possibly due to its high ionization energy (10.4 eV), barely below the energy of the ionization light. The possibility that atomic S is present in the molecular beam, however, cannot be excluded. Moreover, we note that in the past sulfur allotropes larger than S_8_ have also been suggested to be stable, but that these were not observed here^30^. The present experimental distribution is dominated by the octasulfur allotrope S_8_, likely because the precursor sulfur powder may be composed largely of α-sulfur, formed by stacks of S_8_ units. Supplementary Fig. 2 provides the infrared spectrum of solid α-sulfur, supporting this idea. We note that fragmentation of S_8_ induced by the photoionization is unlikely, given that the sum of ionization and fragmentation energies, discussed later in this contribution, is higher than 10.5 eV.

**Fig. 1:**
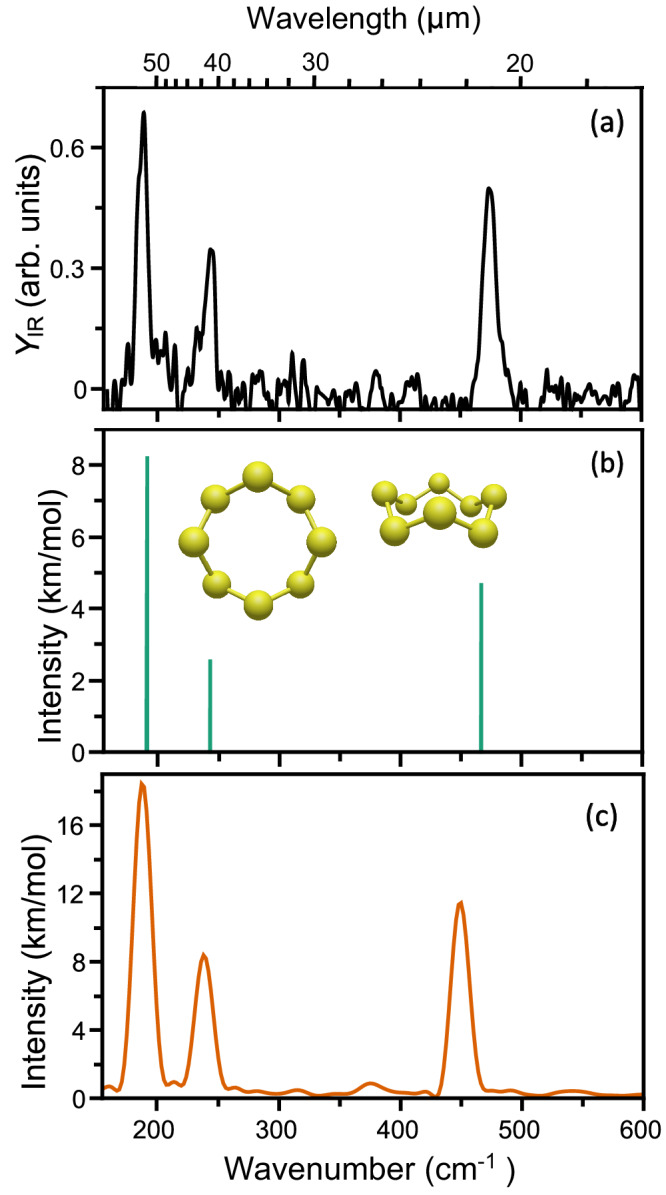
Infrared spectrum of neutral S_8_. **a** Experimental far-infrared spectrum of gas-phase neutral S_8_. **b** Harmonic vibrational modes of S_8_, calculated by density functional theory using the geometry shown as inset, where two views of it are presented. **c** Far-infrared spectrum of S_8_ computed using molecular dynamics simulations at 50 K. Source data are provided as a Source Data file.

The far-infrared spectrum of neutral S_8_ in the 150–600 cm^−1^ (67–17 µm) spectral range is recorded via infrared photodissociation spectroscopy, utilizing the free-electron laser FELIX (Nijmegen, The Netherlands)^31^. The spectrum, presented in Fig. 1a, is composed by registering fragmentation of S_8_ into both S_5_ and S_6_. It shows three clear bands, centered at 187, 242 and 474 cm^−1^ (53.5, 41.3 and 21.1 µm).

Because this experiment relies on the absorption of more than one photon to achieve fragmentation (with the condition that the energy carried by the photons exceeds the fragmentation energy) and we cannot establish how many photons are absorbed per infrared pulse, the experiment does not allow to obtain absolute absorption cross-sections. Nevertheless, the relative absorption cross-sections are crucial to benchmark computational methods. Previous computational studies exploring the potential energy surface of S_8_ reported a crown-shaped ring geometry with singlet spin multiplicity as the putative ground state^32^. Here, we show the harmonic vibrational frequencies of S_8_ for such a geometry (Fig. 1b). The computed vibrational bands at 191, 243 and 467 cm^−1^ agree remarkably well with the experimental spectrum, both in line positions and in relative intensities. Because of the high D_4d_ symmetry of S_8_, the modes at 191 and 467 cm^−1^ are doubly degenerate, whereas the mode at 241 cm^−1^ is non-degenerate. Normal mode vectors are depicted in Supplementary Fig. 3. This high symmetry means that S_8_ has no permanent dipole moment, rendering it invisible to microwave spectroscopy.

The spectral bandwidths of the three observed bands (full-width at half-maximum, FWHM) are close to 10 cm^−1^, and therefore larger than the IR laser bandwidth of FELIX of 1–2 cm^−1^ at these wavelengths. The observed bandwidths could result from a broadening effect innate to the requirement to absorb > 20 IR photons to reach the fragmentation threshold. To test an alternative scenario of dynamical broadening at finite-temperatures, an ab-initio Born–Oppenheimer molecular dynamics (BOMD) simulation was performed. From such simulations, an IR spectrum can be obtained that includes the intrinsic vibrational linewidths, and directly comprises anharmonic effects. The temperature in previous molecular beam studies in the same experimental instrument was shown to range from 40 to 50 K^33^, so we took 50 K as an upper limit here. The result of the 5 ps, 0.5 fs step simulation is shown in Fig. 1c. This simulated spectrum also reproduces the three main bands of S_8_, with only a slightly poorer agreement in band positions than the density functional theory calculation. The dynamics simulations show that the width of these bands is intrinsically larger than the bandwidth of FELIX, as a consequence of the shape fluctuations of S_8_ at 50 K. They simultaneously demonstrate that at 50 K, S_8_ is stable, with only small shape fluctuations of the ground-state geometry, excluding fragmentation or isomeric changes. Finally, smaller features between the main bands are predicted, providing a possible agreement with weaker modes detected close to the experimental noise level. These intrinsic widths of the bands will complicate astronomical observations of free S_8_, as discussed later.

### Destruction pathways of neutral sulfur allotropes

**Fig. 2:**
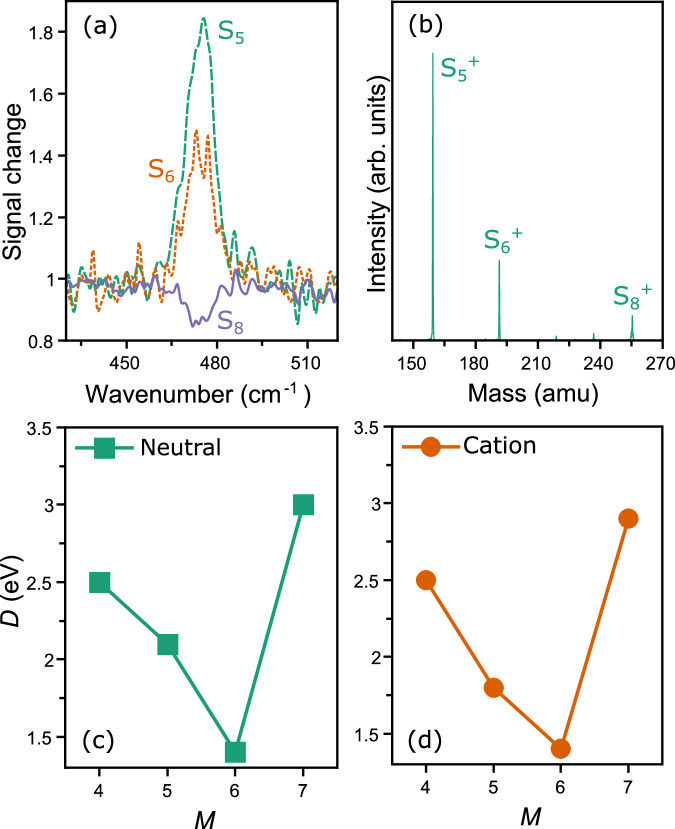
Fragmentation of sulfur clusters. **a** Wavenumber dependence of the laser-induced reduction of S_8_ signal compared with the signal increase detected for S_5_ and S_6_. **b** Fragmentation products after the collision induced dissociation of isolated S_8_^+^ clusters. **c**, **d** Density functional theory calculated fragmentation energies of S_8_ and S_8_^+^ fragmenting into S_*M*_ and S_8-*M*_, or S_*M*_^+^ and S_8-*M*_ species, respectively. Source data are provided as a Source Data file.

In addition to the measurement of the far-IR spectrum of S_8_, the experiments allow a determination of its destruction pathways induced by IR absorption. Because the infrared-induced fragmentation mechanism is statistical in nature, fragmentation follows the lowest-energy pathways, and is likely the same which follows after the absorption of UV or visible photons. Observed fragmentation patterns are therefore crucial for astrochemical modelling of molecular abundances, so far relying on general assumptions or computations^30,34^. Figure 2a presents the wavelength-dependent depletion and appearance (the mass-spectral intensity ratios with and without IR laser light) of neutral S_8_, S_6_ and S_5_. As seen from the figure, a decrease in S_8_ signal (corresponding to laser induced fragmentation) coincides with an increase in S_6_ and S_5_ intensities. Such increases are not observed for the S_7_ and S_4_ channels. Therefore, it is shown experimentally that S_8_ fragments via a competition of the channels S_8_ → S_5_ + S_3_ and S_8_ → S_6_ + S_2_. We note that a clear signal increase for S_2_ and S_3_ is not observed, possibly because of the larger recoil energy of smaller fragments, making them more difficult to detect.

To rationalize the observed fragmentation channels for S_8_, thermodynamic fragmentation energies for the possible S_8_ → S_*M*_ + S_8-*M*_ (*M* = 4–7) pathways (Fig. 2c) are calculated. The lowest fragmentation energies are calculated for the channels leading to S_5_ and S_6_ products. Although both are observed in the experiment, we note that the experiment favors S_5_ formation, whereas the fragmentation energy *D* calculated for forming S_6_ is lower. The reliability of the density functional theory calculated fragmentation energies are confirmed by single-point coupled-cluster (CCSD(T)) calculations (Supplementary Fig. S4). The seemingly conflicting findings between experiment and computations can be evaluated further by not only taking thermodynamic, but also kinetic factors in the fragmentation reaction into account. During fragmentation, the system will encounter energy barriers, for instance when S–S bonds are broken. The values in Fig. 2c therefore correspond to lower limits of the energy needed to fragment S_8_. This is explored by calculating the lowest-energy pathway along the potential energy surface describing the S_8_ → S_6_ + S_2_ fragmentation reaction. Supplementary Fig. S5 shows two energy barriers at 2.2 eV above the energy of S_8_, corresponding to the breaking of the two S-S bonds, placing a higher, kinetic energy limit for fragmentation. Further, it should be considered that fragmentation rates are not only dependent on energetics, but also on the associated entropy change favoring pathways towards trimers over dimer loss^35^. Nevertheless, the key observations are the experimentally determined fragmentation pathways S_8_ → S_5_ + S_3_ and S_8_ → S_6_ + S_2_.

We note that the presence of sulfur in the atmospheres of gas giant exoplanets is used as evidence for photochemistry in an atmosphere that is enhanced in metals. This assumed tracer role of sulfur depends strongly on a non-volatile nature of the sulfur reservoir in planet forming disks. Our study reveals the fragmentation pathways of S_8_, which lead to more volatile sulfur allotropes, and hence would increase the volatility of sulfur in disks. If S_8_ is indeed a major reservoir of sulfur in planet forming disks, this would thus imply that sulfur is less reliable as a tracer of the metal content in gas giant atmospheres.

### Destruction pathways of charged sulfur allotropes

Because local surroundings determine the charge state of interstellar species, with both neutrals and ions being currently identified in the ISM^36^, we also investigated negatively and positively charged sulfur allotropes, using a room temperature quadrupole ion trap^37^. Ions were formed through sublimation at 250 °C and ionization in a plasma corona discharge, before being guided to the He filled ion trap. As shown in Supplementary Fig. S6, this preparation method leads primarily to S_8_^+^ in the cationic charge state. For anions, only S_4_^−^ is observed.

Experimental information about the destruction pathways of ionic sulfur allotropes is obtained via collision induced dissociation (CID)^38^, where a specific allotrope is isolated in the ion trap and the products of fragmentation induced by collisions with He gas are characterized. Isolating and fragmenting the S_8_^+^ allotrope leads to the formation of S_5_^+^ and S_6_^+^ products, in line with previous findings^39^. This is shown in the mass spectrum of Fig. 2b, where upon collision induced dissociation the intensity of the isolated S_8_^+^ decreases, with a concomitant increase in the S_5_^+^ and S_6_^+^ channels. Therefore, S_8_^+^ follows the S_8_^+^ → S_5_^+^ + S_3_ and S_8_^+^ → S_6_^+^ + S_2_ destruction pathways, like neutral S_8_. Here as well, calculations of fragmentation energies (Fig. 2d) show the lowest values for the channels forming cationic S_5_^+^ and S_6_^+^ products, in line with the experimental observations, although the S_5_^+^ channel is again not the thermodynamically favored.

Following the fragmentation of S_8_^+^, the S_6_^+^ and S_5_^+^ products can be further isolated, allowing also characterization of their fragmentation pathways. S_6_^+^ was found to have a unique S_6_^+^ → S_4_^+^ + S_2_ destruction pathway, whereas for S_5_^+^ there is a competition between the S_5_^+^ → S_3_^+^ + S_2_ and S_5_^+^ → S_2_^+^ + S_3_ channels. Finally, S_4_^+^ and S_3_^+^ fragment following S_4_^+^ → S_2_^+^ + S_2_ and S_3_^+^ → S_2_^+^ + S. A summary of calculated fragmentation energies for the different cationic sulfur clusters is presented in Supplementary Fig. 7.

### Far-infrared spectroscopy of ionic S_4_^+^ and S_4_^−^ allotropes

IR induced fragmentation of S_8_^+^ proved unsuccessful, potentially due to a combination of a high fragmentation threshold, medium strength IR cross sections, large heat capacity, and importantly, the presence of He in the ion trap, acting as a heat bath that prevents reaching the required internal energy for fragmentation.

**Fig. 3:**
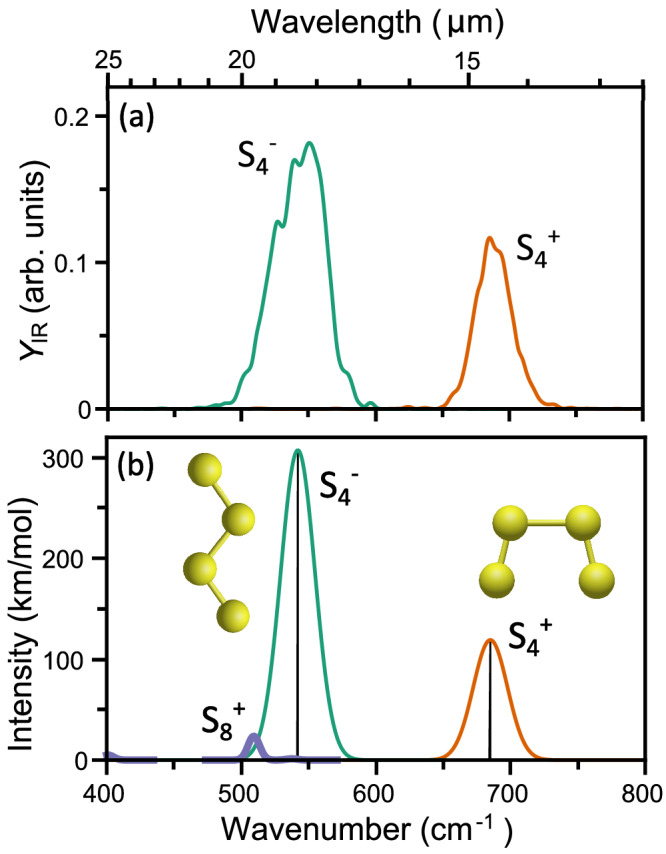
Infrared spectra of charged allotropes. **a** Experimental far-infrared spectra of S_4_^+^ and S_4_^−^, measured in a room temperature ion trap. **b** Density functional theory calculated harmonic vibrational modes of S_4_^+^ and S_4_^−^, using the shown geometries. Transitions are broadened using Gaussian line shapes, broader than the laser spectral profile, to simulate broadening effects in the experiment. For comparison, the calculated vibrational spectrum of S_8_^+^ is also shown. Source data are provided as a Source Data file.

In contrast, for the dominant anionic S_4_^−^ allotrope, as well as for the generated S_4_^+^ fragment, we successfully recorded an IR spectrum. For S_4_^−^ (Fig. 3a) only a single mode is detected in the 400–800 cm^−1^ (12.5–25 µm) spectral range covered in the ion trap experiments, centered at 544 cm^−1^ (18.4 µm). Concomitant calculations yielded a single S_4_^−^ harmonic vibrational mode at 542 cm^−1^ (Fig. 3b), showing a near-perfect match with the experiment. The IR spectrum of S_4_^+^ (Fig. 3a) has, analogous to the S_4_^−^ anion, only a single IR band within the 400–800 cm^−1^ range, centered at 688 cm^−1^ (14.5 µm). A calculation again finds an almost perfect agreement with the experiment, predicting a single vibration at 685 cm^−1^ (Fig. 3b). For comparison, the calculated harmonic vibrational spectrum of S_8_^+^ shows a much lower IR activity than S_4_^+^ and S_4_^−^, a potential reason for the failure to observe infrared-induced fragmentation of S_8_^+^.

### IR absorption cross sections of sulfur allotropes and comparison to comet 67 P/Churyumov-Gerasimenko

Our experiments reveal IR absorption bands for S_*N*_ allotropes concentrated in the 15–22 µm range, with a trend of decreasing wavelength for smaller *N*. Interestingly, this wavelength range coincides with the prominent absorption detected in irradiated laboratory samples that are proposed to be representative of the refractory sulfur-containing organics detected in the comet 67 P/Churyumov-Gerasimenko^40^. In particular, an absorption peak near 21 µm is close to the 21.1 µm band of S_8_. Given that S_2_ and S_3_ desorption products were detected in the warm-up of the laboratory ices, we suggest that the organic residue also contains sulfur allotropes, including S_8_. Because of the relatively low fragmentation energies of only a few eV of most allotropes, the detection of S_2_ and S_3_ in irradiated sulfur bearing ices may well result from larger sulfur allotropes. We note that S_2_ and S_3_ were detected both in comet and in the laboratory residue^40^, and that S_2_ is ubiquitous in cometary comae^41^. This further supports the role of sulfur allotropes as a major sink of sulfur. However, we conjecture that this should be in the form of a distribution, with octasulfur S_8_ as most stable representative, but subject to fragmentation by external fields. Our results support the notion that the presence of S_3_ and S_4_ and a significant fraction of the S_2_ in the comet 67 P/Churyumov-Gerasimenko^12^ are due to fragmentation of S_8_.

Based on our results we can estimate the requirements for detecting sulfur allotropes in the ISM using the JWST MIRI instrument. For neutral S_8_ (Fig. 1a), vibrational modes are experimentally seen at 53.5, 41.3 and 21.1 µm. Of these, only the 21.1 µm band lies within the accessible spectral range^42^ and therefore, could be targeted for detecting S_8_ in space. A key question, however, is the absolute cross section of such a band. Given the excellent agreement between the experimental infrared spectrum and the computed vibrational frequencies and relative intensities presented here, we propose the computational results as a reliable source for deducing the cross sections. Under the assumption of a rigid S_8_ structure, the instrument resolution limited absorption cross-sections for the 21.1 µm band increase from 2.5·10^−19^ to 8.0·10^−19^ cm^2^ when reducing the temperature from 40 to 1 K due to the reduction in population of excited rotational states. Of course, a flexible nature of S_8_, as inferred from the molecular dynamics simulations, lowers these values. We have simulated IR profiles of JWST/MIRI observations, indicating that the direct detection of cold gas phase S_8_ in molecular clouds is challenging, based on the required S/N ratio of the 21.1 µm band in the IR profiles, under the assumption that a significant fraction of the sulfur content goes into S_8_. More details are provided in the Supplementary Figs. 8 and 9. Furthermore, we note that other allotropes have much larger cross sections than S_8_, such as S_8_^+^ with σ = 2.6·10^−18^ cm^2^ for the predicted band at 19.7 µm, S_4_ with σ = 1.4·10^−17^ cm^2^ for the predicted band at 15.4 µm, S_4_^+^ with σ = 1.4·10^−17^ cm^2^ for the measured band at 14.5 µm, and S_4_^−^ with σ = 3.7·10^−17^ cm^2^ for the measured band at 18.4 µm (values calculated at 5 K). A list of cross sections calculated for different sulfur allotropes is presented in Supplementary Table 1. The possibility of detecting other allotropes of course still depends on their abundance in the ISM.

### Final remarks

The IR spectral properties and photostability of neutral S_8_ in the cold and isolated conditions of a molecular beam were investigated, underlying its possible presence in cold interstellar environments. Its far-infrared signature reveals a characteristic mode at 21.1 µm, within reach by JWST, as well as clear bands at 41.3 and 53.5 µm. Computations of the IR spectrum of S_8_, as well as those of S_4_^+^ and S_4_^−^, measured in a room-temperature ion trap, reveal a near-perfect agreement with experimental results. These benchmark results for the calculations lend credibility to future computational work on other sulfur allotropes. For both neutral S_8_ and its cationic and anionic counterparts, the destruction pathways demonstrate a pronounced stability consistent with calculated thermodynamic fragmentation energies >1.5 eV that with inclusion of kinetic barriers likely exceed 2 eV. Our data allow direct tests of the presence of sulfur allotropes in space and in laboratory samples studied with infrared spectroscopy. While the direct detection of S_8_ in space is challenging, the presence of S_2_, S_3_ and S_4_ are signposts of the fragmentation of S_8_, strengthening the evidence that S_8_ is a major sink of sulfur in space. The measured IR spectra, together with the observed fragmentation chemistry, provide a key piece for solving the long-standing Sulfur Depletion Puzzle^43^.

## Methods

### Molecular beam experiments on neutral clusters

Neutral sulfur clusters, S_*N*_ (*N* = 2–8), are formed by laser desorption of sulfur powder (Sigma-Aldrich; 99.98%) mixed with carbon black in a nearly 1:1 ratio. The mixture is pressed on a graphite sample bar that is excited by a mildly focused Nd:YAG laser (1064 nm, 10 Hz, 1 mJ/pulse), while being translated at a fixed speed. The desorbed sulfur is entrained in the expansion of a pulse of argon, released from a pulsed valve operated at a backing pressure of 2 bar, forming a supersonic beam. Collisional cooling with argon thermalizes the entrained sulfur species to typical temperatures of 50 K under these conditions. Support of this assumption is given by comparisons with molecular dynamics simulations. The formed molecular beam is collimated by a 2 mm skimmer before it enters the extraction region of a reflectron time-of-flight mass spectrometer, with a typical mass resolution of *m*/Δ*m* = 2500 at 256 amu (S_8_ mass). The neutral sulfur clusters are probed after ionization with 118 nm (10.5 eV) laser light, generated by frequency tripling the third harmonic of a Nd:YAG laser (355 nm, 10 Hz, 18 mJ/pulse) in a Xe/Ar cell (20 mbar of Xe in 150 mbar of Ar). A scheme of the experimental setup is presented in ref. ^44^.

The infrared spectrum of S_8_ is measured via IR photofragmentation spectroscopy. For this, the molecular beam is illuminated by IR light produced by the free-electron laser FELIX (Nijmegen, The Netherlands). The laser and molecular beams are counter propagating, and IR illumination with a single FELIX pulse (7 µs, 50-100 mJ) takes place roughly 300 µs before ionization. Upon resonant excitation with a vibrational mode of S_8_, multiple-photon excitation heats the cluster until it overcomes the fragmentation threshold, leading to fragmentation. By running FELIX at 5 Hz, half the molecular beam repetition rate, successive mass spectra with and without IR light are recorded allowing for correction of long-term source fluctuations. Resonant absorption is identified from mass-spectral signal depletion of S_8_, in addition to the signal increase of S_5_ and S_6_. This also allows to identify the fragmentation channels of S_8_. The infrared spectrum of S_8_ is expressed in the IR yield

1 $${Y}_{{{{{{\rm{IR}}}}}}}\left(\nu \right)=-{{{{{\mathrm{ln}}}}}}\left(\frac{B\left(\nu \right)}{{B}_{0}}\right)/E\left(\nu \right)$$

with *B(ν)* and *B*_0_ the branching ratio with FELIX excitation at frequency *ν* and without it, respectively. *E(ν)* corresponds to the IR laser pulse energy. *B* is defined as:

2 $$B=\frac{{I}_{8}}{{I}_{5}+{I}_{6}+{I}_{8}}$$

with *I*_*N*_ the signal intensity for allotrope S_*N*_. To obtain the spectra, FELIX is scanned in the 150–600 cm^−1^ (67–17 µm) spectral range, in steps of 1 cm^−1^. The FELIX spectral bandwidth is optimized to have at maximum a standard deviation of 0.5% of the central wavelength.

### Ion trap experiments on cationic clusters

The infrared spectra of S_4_^+^ and S_4_^−^ ions are measured at room temperature on a Bruker amaZon quadrupole ion trap, modified to have optical access to the trapped ions. Details of the experimental setup can be found in ref. ^37, which^ has been used in the past to record infrared spectra of astrochemically relevant molecules, such as fullerene-derivatives, showing good correspondence with astronomical observation^45^. Sulfur powder is used as precursor in an atmospheric pressure ionization source equipped with a direct insertion probe, where the material is placed at the tip of a glass tube heated to 250 °C. After sublimation, the material is ionized in the plasma of a corona discharge, using a potential difference between the end plate and the capillary of 4000 V and a corona current of 6000 nA. This process leads to the formation of S_8_^+^ cations or S_4_^−^ anions, which enter the radio-frequency ion trap where they can be mass-isolated. The IR spectrum of S_4_^−^ is obtained by irradiating the trapped ions with a single FELIX pulse after which the fragmentation yield is derived from the mass spectrum by monitoring the intensity of S_4_^−^ and S_2_^−^ anions. S_8_^+^ is dissociated by collisions with He gas via collision-induced dissociation (CID). Mass-isolation of S_6_^+^ followed by CID produced S_4_^+^, which was mass-isolated and irradiated with 5 pulses of FELIX. In this case, the IR induced fragment ion was S_2_^+^. The IR induced fragmentation yield was linearly corrected for the IR laser pulse energy^46^.

### Density functional theory calculations

The vibrational modes of sulfur allotropes are obtained from density functional theory (DFT) calculations, performed with the ORCA 5.03 software package^47^. The Def2-TZVPP basis set is employed, in addition to dispersion corrections to total energies via the D3BJ method. Calculations are performed with the “verytight” convergency criteria for the SCF cycles and the geometry optimizations, as implemented in ORCA. An initial benchmark analysis of different exchange-correlation functionals was conducted, with harmonic vibrational modes compared with the measured infrared spectrum of S_8_. This comparison is presented in Supplementary Fig. 10 and involves calculations employing the PBE (GGA), TPSS (meta-GGA), PBE0 and B3LYP (hybrids), CAM-B3LYP (long-range separated hybrid) and B2PLYP (double hybrid) functionals. While all functionals yield harmonic infrared spectra closely resembling the experimental spectra, B3LYP is selected for all computations presented here, given its better match with the relative intensities in the experiment. The geometries employed for the calculations are obtained from literature^48^, and re-optimized here (Supplementary Fig. 11). Vertical ionization energies (*IE*_*N*_) of the neutral S_*N*_ clusters are also computed, using Eq. (3), with *E* the zero-point corrected energy of the cluster within brackets. In this case, single-point calculations for S_*N*_^+^ are performed on the optimized geometry of the corresponding S_*N*_ cluster, to account for a vertical transition.

3 $${{IE}}_{N}=E\left({{{{{{\rm{S}}}}}}}_{N}\right)-E\left({{{{{{{\rm{S}}}}}}}_{N}}^{+}\right)$$

The dissociation energy (*D*) of allotrope S_*N*_ or S_*N*_^+^, fragmenting into S_*M*_ + S_*N*-*M*_ or S_*M*_^+^ + S_*N*-*M*_, respectively, is computed via Eqs. (4) or (5),

4 $$D=E\left({{{{{{\rm{S}}}}}}}_{N}\right)-E\left({{{{{{\rm{S}}}}}}}_{M}\right)-E\left({{{{{{\rm{S}}}}}}}_{N-M}\right)$$

5 $$D=E\left({{{{{{{\rm{S}}}}}}}_{N}}^{+}\right)-E\left({{{{{{{\rm{S}}}}}}}_{M}}^{+}\right)-E\left({{{{{{\rm{S}}}}}}}_{N-M}\right)$$

The computed vibrational spectra of all S_*N*_ and S_*N*_^+^ allotropes are presented in Supplementary Figs. 12 and 13.

### Ab initio Born–Oppenheimer molecular dynamics simulations

The infrared spectrum of neutral S_8_ is also constructed from a dynamical picture using Ab initio Born–Oppenheimer molecular dynamics (BOMD) simulations. In this case, the CP2K 6.1 software package^49^ is employed, using the B3LYP functional and the TZV2P-GTH basis set, including D3BJ dispersion corrections. A density cutoff of 400 Ry is fixed, and the simulations run in a vacuum periodic orthorhombic box of 25 Å length. The dynamics starts with the S_8_ geometry determined by DFT, after which a total time of 5 ps is simulated, in steps of 0.5 fs. A canonical (NVT) ensemble is employed, with a temperature of 50 K, constrained through a velocity rescaling thermostat. Molecular dipole moments are evaluated from the maximally localized Wannier function centers, and the infrared spectrum is calculated from these coordinates using the TRAVIS software package^50^.

### Supplementary information

Supplementary Information

Peer Review File

### Source data

Source Data

## Data Availability

All the data supporting this study are available in the article and associated Supplementary Information. Source data are provided with this paper.

## References

[1] Krijt, et al. In Protostars and Planets VII, (eds) Inutsuka, S., Aikawa, Y., Muto, T., Tomida, K. & Tamura, M., ASP conference series. 1031 (2023).

[2] Greenwood, N. N. & Earnshaw, A. *Chemistry of the Elements*. (Elsevier, Oxford, 1997).

[3] Mifsud DV (2021). Sulfur ice astrochemistry: a review of laboratory studies. Space Sci. Rev..

[4] Fuente, A. et al. PDRs4All. IX. Sulfur elemental abundance in the Orion Bar. *A&A* **687**, A87 (2024).

[5] Hily-Blant, P., Pineau des Forêts, G., Faure, A. & Lique, F. Sulfur gas-phase abundance in dense cores. *A&A* **658**, A168 (2022).

[6] Fuente A (2023). Gas phase elemental abundances in molecular cloudS (GEMS) VII. Sulfur elemental abundance. A&A.

[7] Neufeld DA (2015). Sulphur-bearing molecules in diffuse molecular clouds: new results from SOFIA/GREAT and the IRAM 30 m telescope. A&A.

[8] Goicoechea JR (2006). Low sulfur depletion in the Horsehead PDR. A&A.

[9] Laas JC, Caselli P (2019). Modeling sulfur depletion in interstellar clouds. A&A.

[10] Le Gal R (2021). Molecules with ALMA at Planet-forming Scales (MAPS). XII. Inferring the C/O and S/H ratios in protoplanetary disks with sulfur molecules. ApJS.

[11] McClure MK (2023). An Ice Age JWST inventory of dense molecular cloud ices. Nat. Astron..

[12] Calmonte U (2016). Sulphur-bearing species in the coma of comet 67P/Churyumov–Gerasimenko. MNRAS.

[13] Wasson JT, Kallemeyn GW (1988). Compositions of chondrites. RSPTA.

[14] Alexander CMO’D, Wynn JG, Bowden R (2022). Sulfur abundances and isotopic compositions in bulk carbonaceous chondrites and insoluble organic material: Clues elemental isotopic fractionations volatile chalcophiles.. Meteorit. Planet. Sci..

[15] Yoshimura T (2023). Chemical evolution of primordial salts and organic sulfur molecules in the asteroid 162173 Ryugu. Nat. Commun..

[16] Tsai S-M (2023). Photochemically produced SO_2_ in the atmosphere of WASP-39b. Nature.

[17] Dyrek A (2024). SO_2_, silicate clouds, but no CH_4_ detected in a warm Neptune. Nature.

[18] Shingledecker CN (2020). Efficient production of S_8_ in interstellar ices: the effects of cosmic-ray-driven radiation chemistry and nondiffusive bulk reactions. ApJ.

[19] Cazaux S (2022). Photoprocessing of H_2_S on dust grains-Building S chains in translucent clouds and comets. A&A.

[20] Mifsud DV (2022). Energetic electron irradiations of amorphous and crystalline sulphur-bearing astrochemical ices. Front. Chem..

[21] Aponte JC (2023). PAHs, hydrocarbons, and dimethylsulfides in Asteroid Ryugu samples A0106 and C0107 and the Orgueil (CI1) meteorite. EPS.

[22] Francés-Monerris A (2022). Photochemical and thermochemical pathways to S_2_ and polysulfur formation in the atmosphere of Venus. Nat. Commun..

[23] Way M (2023). Synergies between venus & exoplanetary observations. SSR.

[24] Jones RO, Ballone P (2003). Density functional and Monte Carlo studies of sulfur. I. Structure and bonding in S_n_ rings and chains (n=2-18). J. Chem. Phys..

[25] Trofimov BA, Sinegovskaya LM, Gusarova NK (2009). Vibrations of the S–S bond in elemental sulfur and organic polysulfides: a structural guide. J. Sulphur Chem..

[26] Steudel R, Schuster F (1978). Vibrational spectra, force constants and thermodynamic functions of cycloheptasulfur, S_7_. J. Mol. Struct..

[27] Zysman-Colman E, Leste-Lassere P, Harpp DN (2008). Probing the chemistry of rare sulfur allotropes: S_9_, S_12_ and S_20_. J. Sulphur Chem..

[28] Thorwirth S (2005). Rotational spectroscopy and equilibrium structures of S_3_ and S_4_. J. Chem. Phys..

[29] Lai, T. S.-Y. et al. GOALS-JWST: Tracing AGN Feedback on the Star-forming Interstellar Medium in NGC 7469. *ApJL,* 941, L36 (2022).

[30] Fedyaeva M, Lepeshkin S, Oganov AR (2023). Stability of sulfur molecules and insights into sulfur allotropy. Phys. Chem. Chem. Phys..

[31] Yatsyna V (2016). Infrared action spectroscopy of low-temperature neutral gas-phase molecules of arbitrary structure. Phys. Rev. Lett..

[32] Cho E, Pratik SM, Pyun J, Coropceanu V, Brédas JL (2022). Ring-to-chain structural relaxation of elemental sulfur upon photoexcitation. ACS Mater. Lett..

[33] Lemmens AK (2023). Wetting of a hydrophobic surface: Far-IR action spectroscopy and dynamics of microhydrated naphthalene. J. Phys. Chem. Lett..

[34] Shingledecker CN, Herbst H (2018). A general method for the inclusion of radiation chemistry in astrochemical models. Phys. Chem. Chem. Phys..

[35] Ferrari P, Hansen K, Lacinbala O, Janssens E, Lievens P (2023). Fragmentation channels of non-fullerene cationic carbon clusters. Phys. Chem. Chem. Phys..

[36] McGuire BA (2022). 2021 census of interstellar, circumstellar, extragalactic, protoplanetary disk, and exoplanetary molecules. ApJS.

[37] Martens J, Berden G, Gebhardt CR, Oomens J (2016). Infrared ion spectroscopy in a modified quadrupole ion trap mass spectrometer at the FELIX free electron laser laboratory. Rev. Sci. Instrum..

[38] Koyasu K, Ohtaki T, Hori N, Misaizu F (2012). Isomer-resolved dissociation of small carbon cluster cations, C7^+^–C10^+^. Chem. Phys. Lett..

[39] Fales HM, Pu Q-L, Mason RT, Pannell LK (1991). The ion trap mass spectrum of sulfur. Int. J. Mass Spectrom..

[40] Mahjoub A (2023). Complex organosulfur molecules on comet 67P: evidence from the ROSINA measurements and insights from laboratory simulations. Sci. Adv..

[41] Bockelée-Morvan, D., Crovisier, J., Mumma, M. J. & Weaver, H. A. The composition of cometary volatiles. *in Comets II*, (2004).

[42] Armus L (2023). GOALS-JWST: mid-infrared spectroscopy of the nucleus of NGC 7469. ApJL.

[43] Ruffle DP, Hartquist TW, Caselli P, Williams DA (1999). The sulphur depletion problem. MNRAS.

[44] Ferrari P, Lemmens AK, Redlich B (2024). Infrared bands of neutral gas-phase carbon clusters in a broad spectral range. Phys. Chem. Chem. Phys..

[45] Palotás J, Martens J, Berden G, Oomens J (2020). The infrared spectrum of protonated buckminsterfullerene C_60_H. Nat. Astron..

[46] Berden G, Derksen M, Houthuijs KJ, Martens J, Oomens J (2019). An automatic variable laser attenuator for IRMPD spectroscopy and analysis of power-dependence in fragmentation spectra. Int. J. Mass Spectrom..

[47] Neese F, Wennmohs F, Becker U, Riplinger C (2020). The ORCA quantum chemistry program package. J. Chem. Phys..

[48] Jin Y (2015). Geometries, stabilities and fragmental channels of neutral and charged sulfur clusters: S_n_^Q^ (n = 3–20, Q = 0, ±1). Phys. Chem. Chem. Phys..

[49] Kühne TD (2020). CP2K: An electronic structure and molecular dynamics software package-Quickstep: Efficient and accurate electronic structure calculations. J. Chem. Phys..

[50] Brehm M, Kirchner B (2011). TRAVIS-a free analyzer and visualizer for Monte Carlo and molecular dynamics trajectories. J. Chem. Inf. Model..

