# Supplementary material for: Laboratory infrared spectra and fragmentation chemistry of sulfur allotropes
Source: Nat Commun. 2024 Jul 15;15:5928. doi: 10.1038/s41467-024-50303-2 (PMC11251068; doi:10.1038/s41467-024-50303-2)
Supplement: Supplementary file 1 — Supplementary Information [file 41467_2024_50303_MOESM1_ESM.pdf]

Supplementary Information

Laboratory infrared spectra and fragmentation chemistry of sulfur allotropes

Piero Ferrari[1,*], Giel Berden[1], Britta Redlich[1], Laurens B. F. M. Waters[2] & Joost M. Bakker[1]

[1] Radboud University, Institute for Molecules and Materials, FELIX Laboratory, Nijmegen, The Netherlands
[2] Department of Astrophysics, IMAPP, Radboud University, Nijmegen, Netherlands

Content

1. Typical mass spectrum of neutral S_N
2. Infrared spectrum of sulfur powder
3. Normal mode vectors of neutral S_8
4. Dissociation energies calculated with CCSD(T)
5. Potential energy surface along the $S_8 \rightarrow S_6 + S_2$ dissociation pathway
6. Mass spectrum of cationic S_N^+ allotropes
7. Dissociation energies of cationic sulfur allotropes
8. IR cross section and astronomical detections
9. Benchmark of exchange-correlation functional in the DFT calculation of IR spectra
10. Geometries of S_N and S_N^+ allotropes
11. DFT calculated infrared spectra of S_N and S_N^+ allotropes
12. Supplementary References

1. Typical mass spectrum of neutral S_N

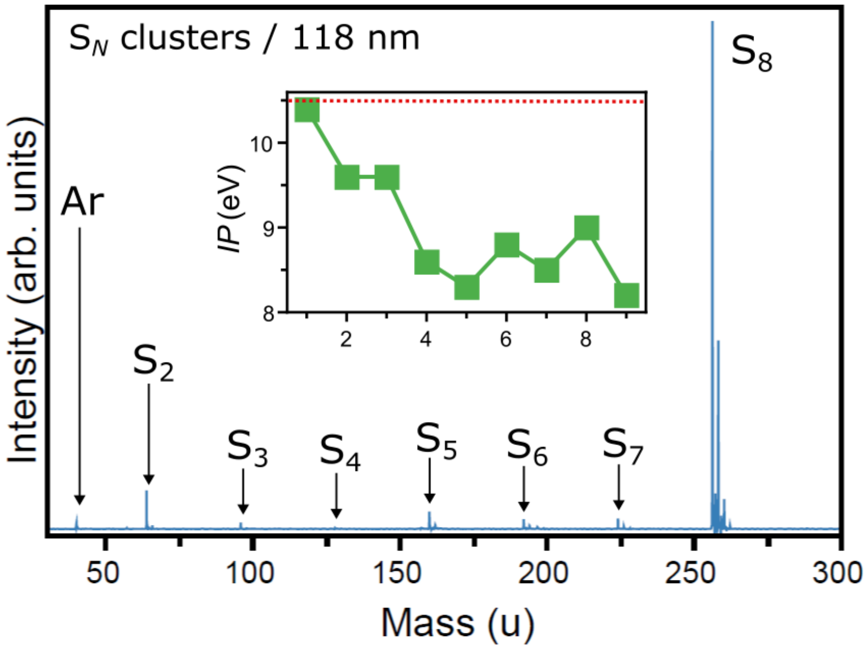

Supplementary Figure 1. Mass spectrum of neutral S_N clusters in a molecular beam, photoionized with 118 nm laser light. The naturally abundant S isotopes are reflected in the satellite peaks for each species. The inset presents DFT calculated vertical ionization energies, with the energy used for ionization highlighted by the red line.

2. Infrared spectrum of sulfur powder

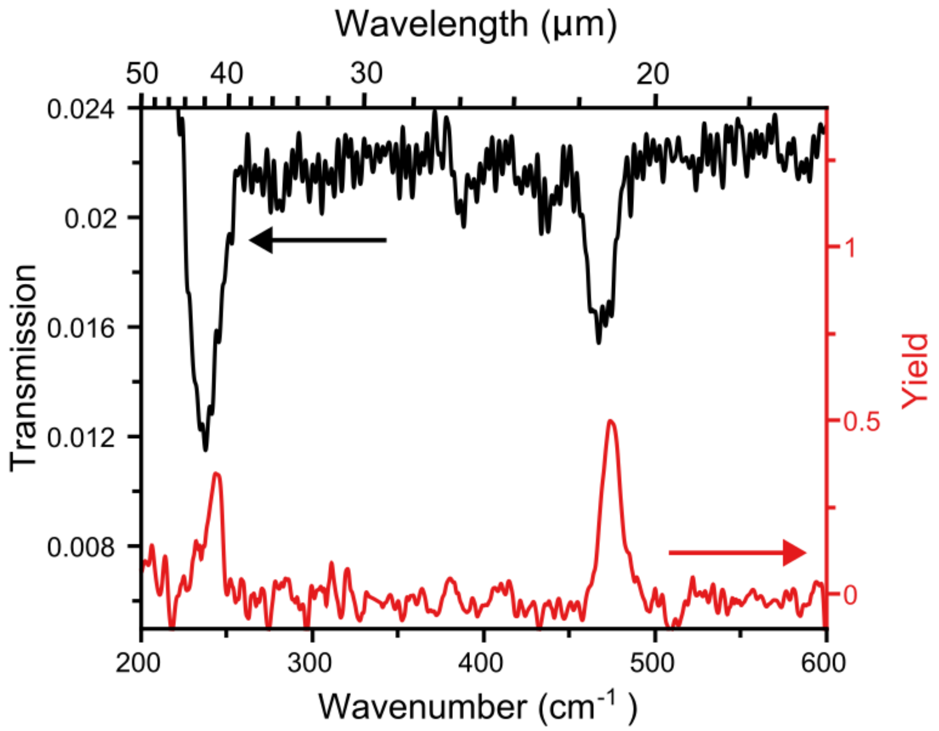

Supplementary Figure 2. Infrared spectrum of sulfur powder (top black), recorded via Fourier-transform infrared (FTIR) spectroscopy in the 220-600 cm^{-1} range. For comparison, the infrared spectrum of S_8 measured in a molecular beam (same plot as in Fig. 1a of main article) is depicted (bottom red). Clearly, the infrared spectrum of the sulfur powder presents the two main features of gas-phase S_8, although with a small red-shift. Interestingly, the FTIR spectrum resolves two smaller features around 400 cm^{-1}, which may also be present in the gas-phase S_8 curve. The clear correspondence between the spectra of sulfur powder and gas-phase S_8 strongly suggests that the powder is composed to a large extent of α-sulfur.

3. Normal mode vectors of neutral S_8

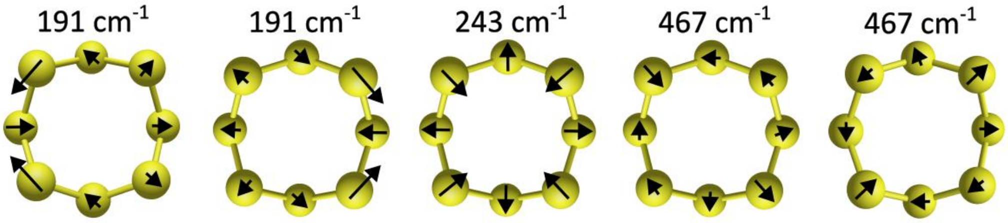

Supplementary Figure 3. Normal mode vectors of neutral S_8. Representation of the infrared active vibrational modes of neutral S_8.

4. Dissociation energies calculated with CCSD(T)

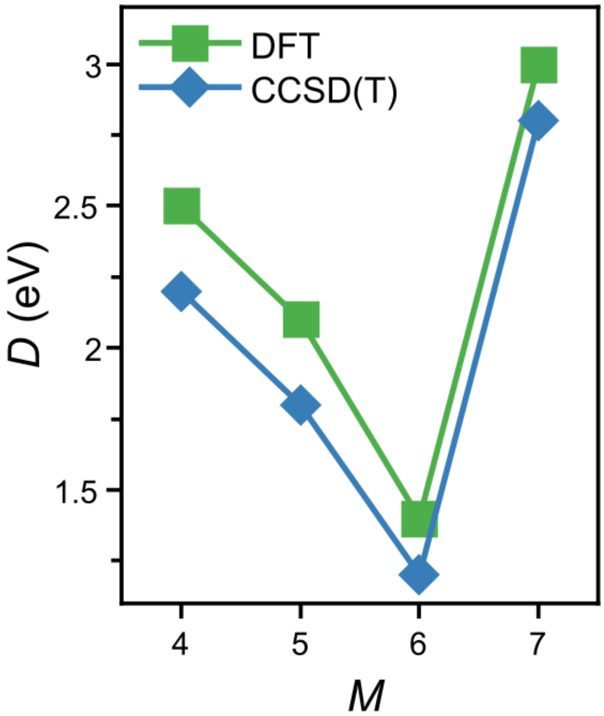

Supplementary Figure 4. Dissociation energies calculated with CCSD(T). Comparison of dissociation energies for the different destruction pathways of S_8, calculated by DFT (same as in the main article), and with coupled-cluster theory CCSD(T), using single-point calculations with the geometries optimized by DFT. In both cases, the same basis set is employed (def2-TZVPP), as well as the same SCF convergency criteria and integration grid size. While both methods show a slight off set in the dissociation energies, the trend with size is well reproduced.

5. Potential energy surface along the $S_8 \rightarrow S_6 + S_2$ dissociation pathway

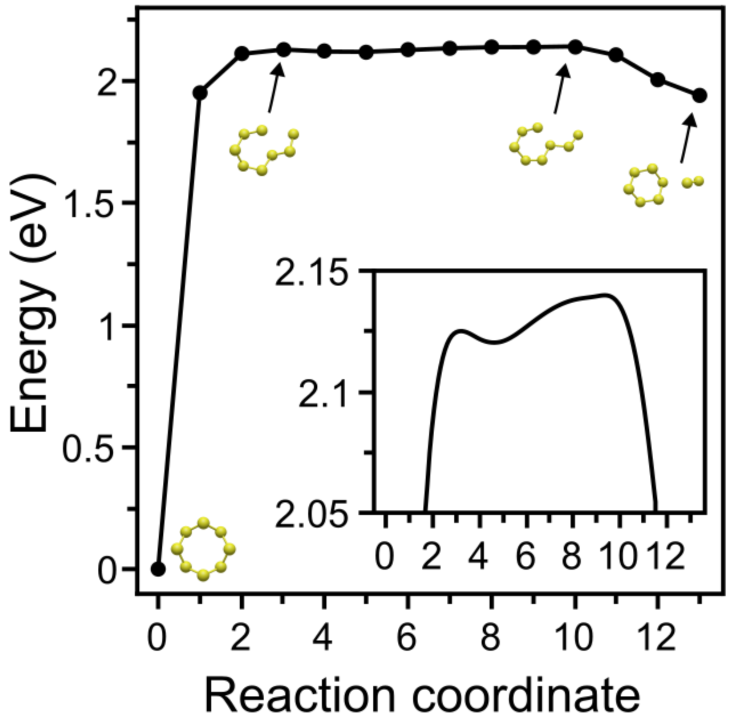

Supplementary Figure 5. Potential energy surface along the $S_8 \rightarrow S_6 + S_2$ dissociation pathway. Lowest-energy pathway along the potential energy surface connecting S_8 and the dissociated S_6 and S_2 allotropes, calculated using the Nudged Elastic Band (NEB) method, implemented in ORCA. For this calculation, the B3LYP functional was employed, together with the Def2-SVP basis set. Two local maxima are seen in the energy path, corresponding to the two S-S bonds that need to be broken to emit a S_2 dimer. The inset shows a zoom in around the local maxima.

6. Mass spectrum of cationic S_N^+ allotropes

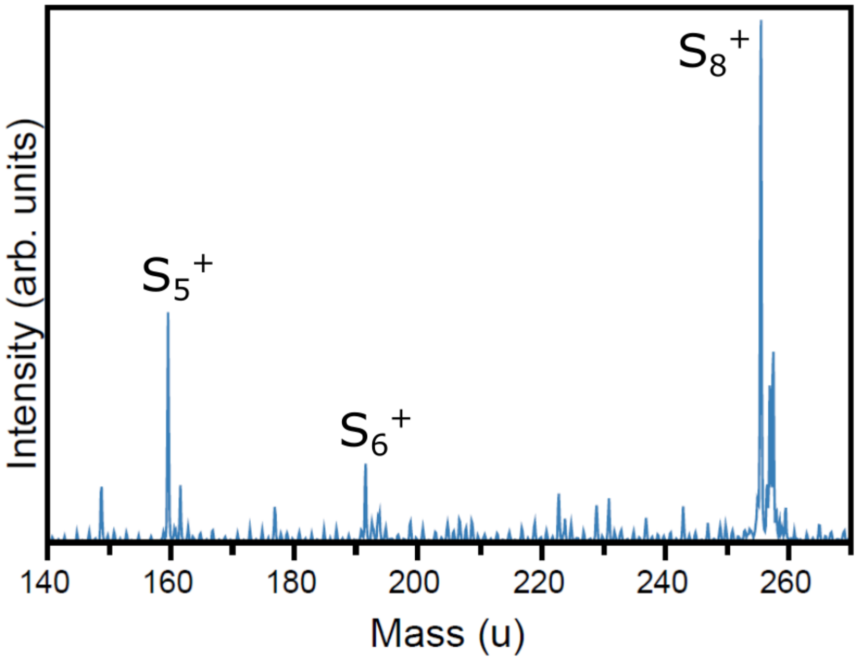

Supplementary Figure 6. Mass spectrum of S_N^+ clusters measured on the Bruker amaZon ion trap at FELIX.

7. Dissociation energies of cationic sulfur allotropes

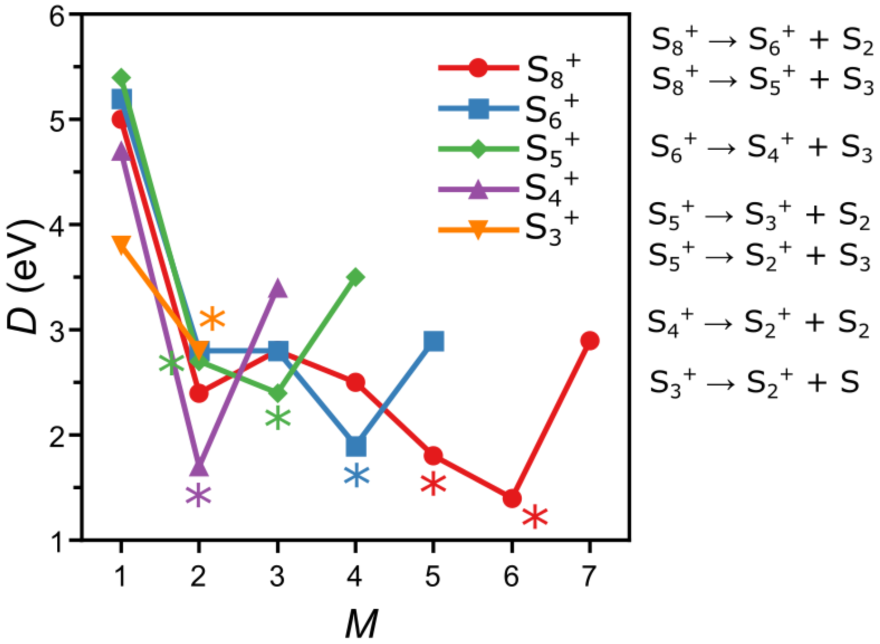

Supplementary Figure 7. Dissociation energies of cationic sulfur allotropes. Dissociation energies of S_N^+ clusters fragmenting into S_M^+ and $S_{N\text{-}M}$ products, calculated by DFT. The experimentally observed dissociation pathways are shown at the right of the plot, and are also highlighted by asterisks in each curve. As seen, the observed pathways always agree with the lowest dissociation energies.

8. IR cross section and astronomical detections

Quantum chemical software typically provide infrared intensities (I) per vibrational mode in units of km/mol, which represent the line-integrated molar cross section. To convert these to cross sections per molecule (σ), we assume that the bands have a Gaussian shape:

$$\sigma(\nu) = \frac{10^5}{WN_A}\sqrt{\frac{2}{\pi}}e^{-2\left(\frac{\nu-\nu_0}{W}\right)^2}I, \qquad (S1)$$

where ν is the frequency in cm^{-1}, ν_0 the band center, W the width of the Gaussian (2 standard deviations), and N_A Avogadro's number. Therefore, an important assumption is the width of the band. We first approximate this width by simulating the rotational substructure using the PGOPHER package, assuming an oblate symmetric top molecule with DFT calculated rotational constants (a = 0.022 cm^{-1}, c = 0.012 cm^{-1}) at temperatures of 1, 5, 10, 20 and 40 K. From these simulations, we obtain a (fitted) Gaussian width, with which the cross sections are obtained. Curves are shown in Figure S14a.

If we take into account the observed spectral width of around 10 cm^{-1} (Fig. 1a of main article), the cross sections per S_8 molecule can be calculated, as depicted in Fig. S14b. For the mode at 21.10 μm, this analysis gives a cross section of $6.2 \cdot 10^{-20}$ cm^2. The 10 cm^{-1} width, however, can result from a combination of the internal temperature of S_8 in the molecular beam, the spectral linewidth of FELIX, and potential broadening effects in the use of IRMPD for measuring the IR spectrum. It is therefore a hard upper limit, resulting in a hard lower limit for the cross sections calculated for Figure S14b. We also provide the cross sections calculated for different sulfur allotropes at 5 K in Table S1. For those values, the most infrared intense vibrational mode is selected.

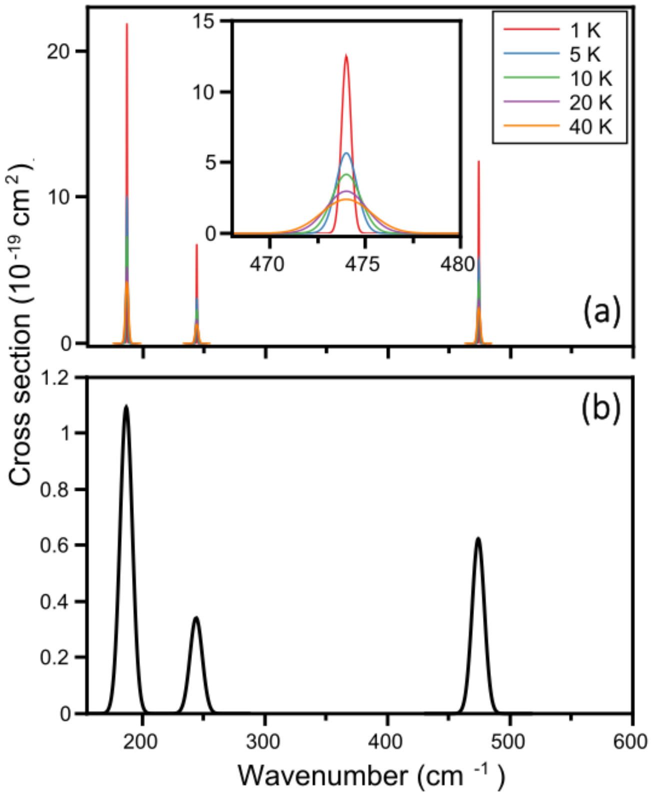

Supplementary Figure 8. IR cross sections. Cross section per S_8 molecule calculated from the DFT simulated integrated infrared intensities and assuming Gaussian shapes for each infrared bands. In panel (a), the results for different temperatures are presented, with a zoom in on the 21.10 μm band. In panel (b), instead, the curve constructed with widths of 10 cm^{-1} are shown.

Supplementary Table 1. Cross section at 5 K for different sulfur allotropes, in their neutral and ionic states. The most intense vibrational mode is selected.

**Allotrope**	**Band center (μm)**	**Cross section (cm^2)**
S_3	14.59	$1.53 \cdot 10^{-17}$
S_4	15.43	$1.41 \cdot 10^{-17}$
S_5	23.53	$1.64 \cdot 10^{-18}$
S_6	21.82	$9.23 \cdot 10^{-19}$
S_7	19.08	$1.08 \cdot 10^{-18}$
S_8	21.10	$5.67 \cdot 10^{-19}$
S_3^+	47.93	$1.22 \cdot 10^{-17}$
S_4^+	14.55	$1.43 \cdot 10^{-17}$
S_5^+	27.49	$6.71 \cdot 10^{-19}$
S_6^+	21.91	$1.19 \cdot 10^{-18}$
S_7^+	29.71	$1.27 \cdot 10^{-18}$
S_8^+	19.68	$2.59 \cdot 10^{-18}$
S_3^-	18.01	$1.89 \cdot 10^{-17}$
S_4^-	18.38	$3.68 \cdot 10^{-17}$
S_8^-	19.46	$4.61 \cdot 10^{-18}$

We estimate the expected spectroscopic signature of S_8 molecules in cold interstellar clouds by modeling a pencil beam of light from a background source of arbitrary flux, that passes through a molecular cloud in which gas and dust absorb the light from the background star. We assume no emission from the cloud itself. The background star is assumed to radiate as a black body. The flux observed on Earth then is:

$$F(\text{obs}, \nu) = F(\text{star}, \nu) \cdot e^{-\tau(\nu)}, \qquad (S2)$$

where $\tau(\nu)$ has two components, $\tau(\text{dust})$, which is the optical depth due to the dust particles along the line of sight, and $\tau(S_8)$, corresponding to the optical depth of the S_8 molecules. To estimate the dust optical depth, we use the extinction law derived by Ref. [2], which provides the dust extinction given a column density of atomic hydrogen for lines of sight representative of the diffuse interstellar medium. We note that in dense molecular clouds the grain size distribution may result in different extinction laws. To estimate the optical depth of the S_8 molecules along the line of sight, we use the cross-sections derived above. We assume a cosmic sulfur abundance of $1.4 \cdot 10^{-5}$ with respect to hydrogen and furthermore assume that half of the available sulfur is in S_8 [3]. The actual abundance of S_8 is not well constrained, and therefore this calculation serves as a rough estimate of the detectability of S_8 in interstellar clouds. Furthermore, we are assuming that the cross-sections of the gas-phase molecules also hold in an interstellar ice. The adopted S_8 abundance is consistent with the known sulfur budget in clouds [4]. To simulate the JWST spectrum, we furthermore assume a signal to noise level of 100, which can be reached using the JWST/MIRI instrument [5] for flux levels of the order of 0.1 Jy. An H_2 column of 5×10^{22} cm^2 was used, corresponding to an extinction in the visual of 35 magnitudes. The resulting model spectra are shown in Figure 15a,b.

In the simulations we have used the in-orbit performance of MIRI and have chosen a detection limit of 3σ of S_8 absorption at 21 μm. The in-orbit performance of MIRI shows that a S/N of 100 is achievable for source fluxes of 100 mJy, as a typical noise level at 21 μm for exposure times of 1-2 hours is about 1 mJy. Just as an example of a bright source, we have assumed a background source at 1 kiloparsec distance emitting as a 20000 K black body and a luminosity of 130.000 solar luminosities, which would correspond to a young OB-type main sequence star. A noise level of 1 mJy and S/N of 100 translate to a 3 per cent absorption band that is minimally required to reach a S/N of 3 of the absorption. This is achieved when the intrinsic width of the feature is similar to the spectral resolution of the MIRI instrument at 21 μm, which we have assumed to be $\lambda/\Delta\lambda = 1000$. We have furthermore assumed that half of the cosmic abundance of sulfur is locked into S_8, based on chemical network models. We have also assumed several intrinsic bandwidths of the S_8 resonance, that are motivated in the text. The detectability of S_8 also depends on the column of interstellar gas and dust in the line of sight. To detect S_8 the optical extinction in magnitudes A_V has to be above about 30 magnitudes to

reach a S/N of 3. Very high extinction values (above 90-100) result in a lower continuum flux, pushing the S/N at 21 µm below 100.

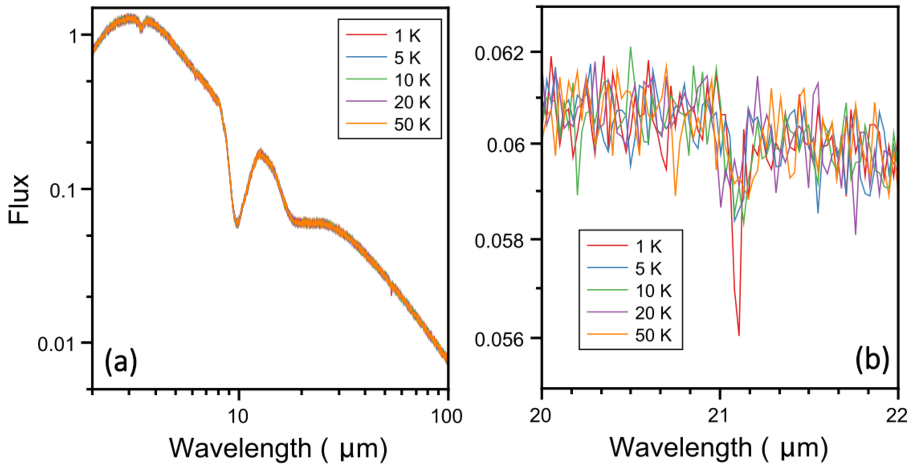

Supplementary Figure 9. Simulated JWST spectra. (a) Simulated JWST spectra at different S_8 temperatures. The deep absorptions are from amorphous silicate dust grains in the line of sight. (b) Zoom in on panel (b), around the 21.10 µm band.

9. Benchmark of exchange-correlation functional in the DFT calculation of IR spectra

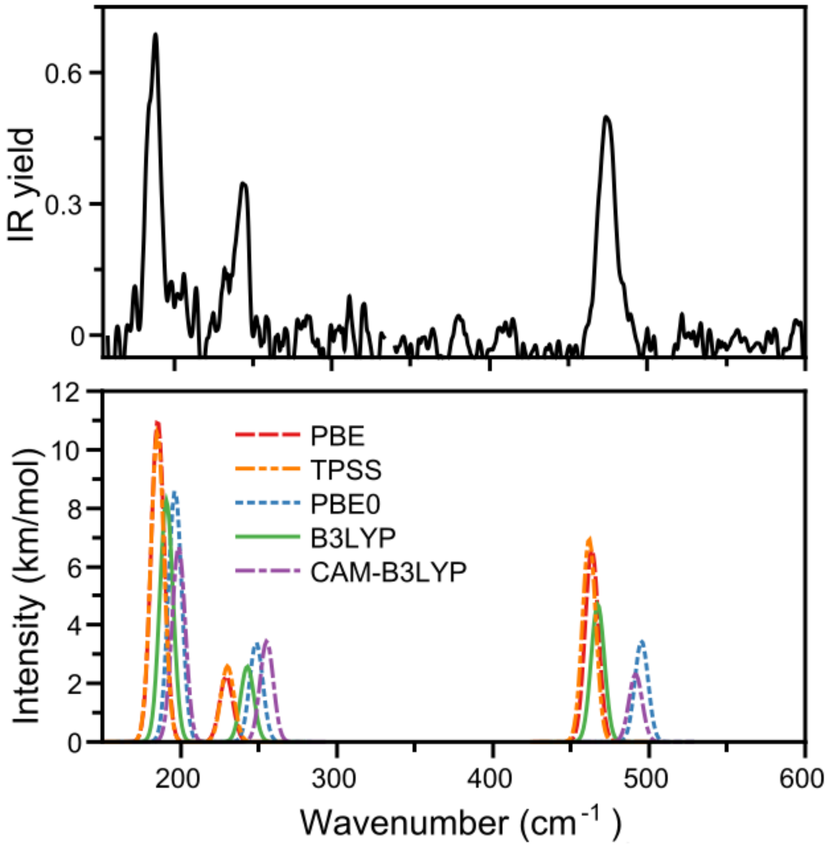

Supplementary Figure 10. Benchmark of exchange-correlation functional in the DFT calculation of IR spectra. Comparison of the measured IR spectrum of S_8 (top) and harmonic vibrational frequencies computed with DFT using different exchange-correlation functionals. In all cases, the Def2-TZVPP basis set is employed, together with D3BJ dispersion corrections.

10. Geometries of S_N and S_N^+ allotropes

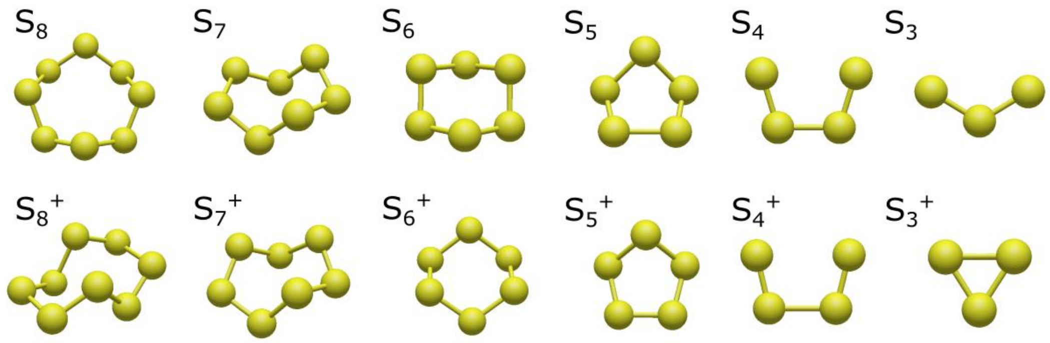

Supplementary Figure 11. Geometries of sulfur allotropes. Ground-state geometries of S_N and S_N^+ allotropes optimized at the DFT level, using the B3LYP functional and def2-TZVPP basis set, in addition to DB3J dispersion correction to the total energy.

11. DFT calculated infrared spectra of S_N and S_N^+ allotropes

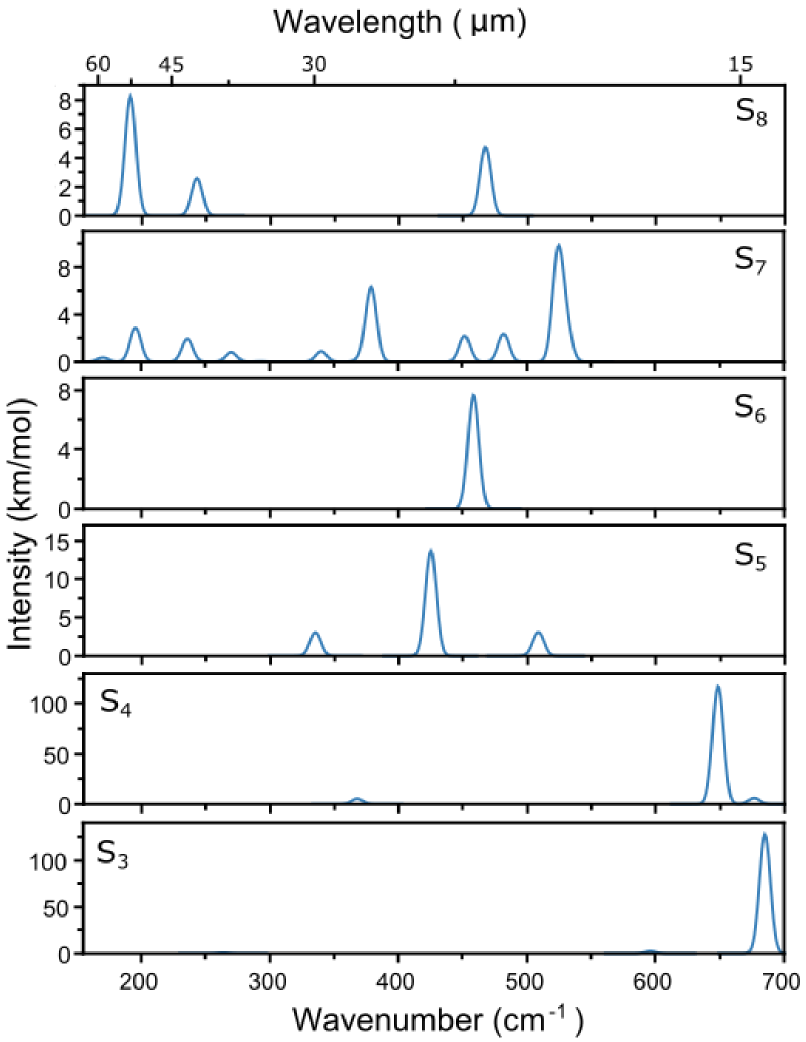

Supplementary Figure 12. Computed IR spectra. DFT-calculated infrared spectra of S_N allotropes, computed at the B3LYP/def2-TZVPP level, including the DB3J dispersion correction to the total energy. Transitions are broadened using Gaussian line shapes with a FWHM of 10 cm^{-1}.

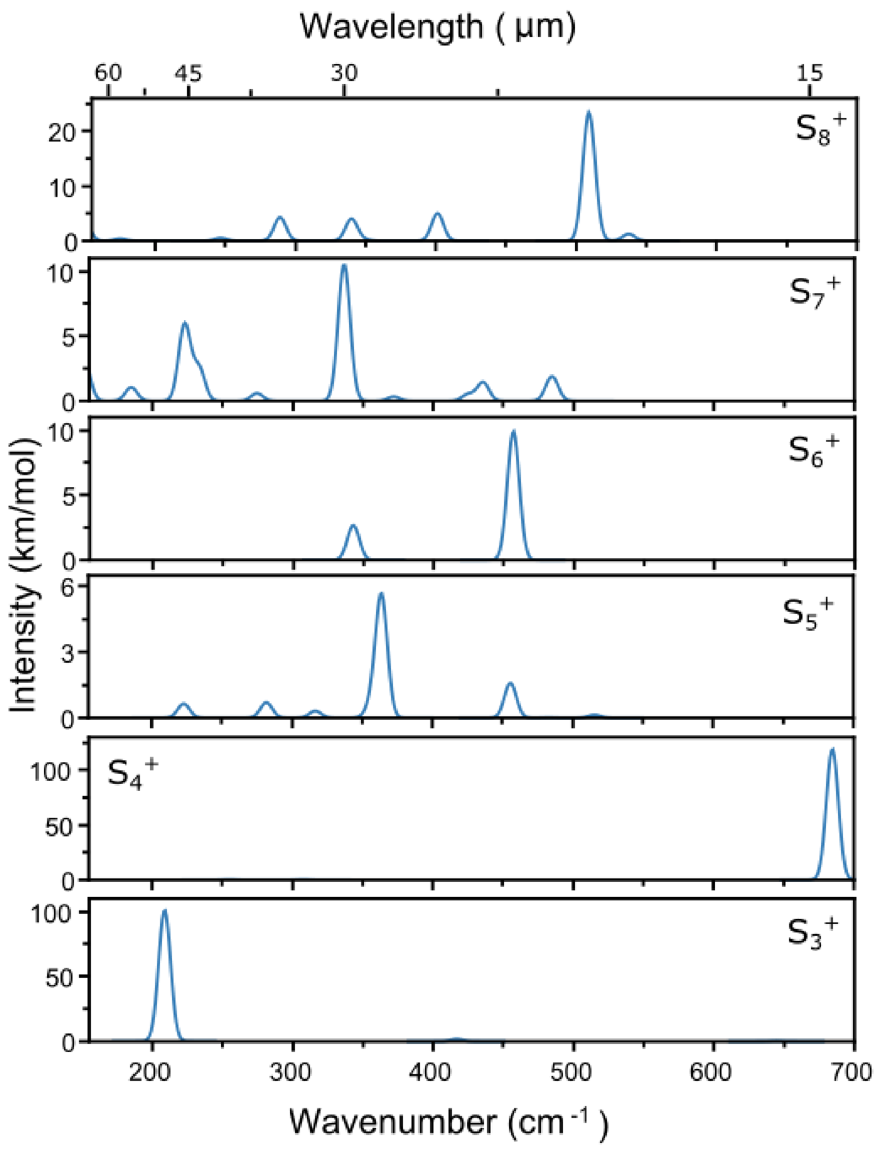

Supplementary Figure 13. Computed IR spectra. DFT-calculated infrared spectra of S_N^+ allotropes, computed at the B3LYP/def2-TZVPP level, including the DB3J dispersion correction to the total energy. Transitions are broadened using Gaussian line shapes with a FWHM of 10 cm^{-1}.

12. Supplementary References

[1] C. M. Western, JQSRT, 186 221-242 (2017)

[2] B. S. Hensley and B. T. Draine, ApJ, 895, 38 (2020).

[3] C. N. Shingledecker, T. Lamberts, J. C. Laas, A. Vasyunin, E. Herbst, J. Kästner and P. Caselli, ApJ, 888, 52 (2020).

[4] M. K. McClure, W. R. M. Rocha, K. M. Pontoppi-dan, N. Crouzet, L. E. U. Chu, E. Dartois, T. Lam-berts, J. A. Noble, Y. J. Pendleton, G. Perotti, D. Qasim, M. G. Rachid, Z. L. Smith, Fengwu Sun, Tra-cy L. Beck, A. C. A. Boogert, W. A. Brown, P. Casel-li, S. B. Charnley, H. M. Cuppen, H. Dickinson, M. N. Drozdovskaya, E. Egami, J. Erkal, H. Fraser, R. T. Garrod, D. Harsono, S. Ioppolo, I. Jiménez-Serra, M. Jin, J. K. Jørgensen, L. E. Kristensen, D. C. Lis, M. R. S. McCoustra, Brett A. McGuire, G. J. Melnick, Karin I. Öberg, M. E. Palumbo, T. Shimonishi, J. A. Sturm, E. F. van Dishoeck, H. Linnartz, Nat. Astron. 7, 431–443 (2023).

[5] G. S. Wright, G. H. Rieke, A. Glasse, M. Ressler, M. García Marín, J. Aguilar, S. Alberts, J. Álvarez-Márquez, I. Argyriou, K. Banks, P. Baudoz, A. Boccaletti, P. Bouchet, J. Bouwman, B. R. Brandl, D. Breda, S. Bright, S. Cale, L. Colina, C. Cossou, A. Coulais, M. Cracraft, W. De Meester, D. Dicken, M. Engesser, M. Etxaluze, O. D. Fox, S. Friedman, H. Fu, D. Gasman, A. Gáspár, R. Gastaud, V. Geers, A. Michael Glauser, K. D. Gordon, T. Greene, T. R. Greve, T. Grundy, M. Güdel, P. Guillard, P. Haderlein, R. Hashimoto, T. Henning, D. Hines, B. Holler, Ö. Hunor Detre, A. Jahromi, B. James, O. C. Jones, K. Justtanont, P. Kavanagh, S. Kendrew, P. Klaassen, O. Krause, A. Labiano, P.-O. Lagage, S. Lambros, K. Larson, D. Law, D. Lee, M. Libralato, J. Lorenzo Alverez, M. Meixner, J. Morrison, M. Mueller, K. Murray, M. Mycroft, R. Myers, O. Nayak, B. Naylor, B. Nickson, A. Noriega-Crespo, G. Östlin, B. O'Sullivan, R. Ottens, P. Patapis, K. Penanen, M. Pietraszkiewicz, T. Ray, M. Regan, A. Roteliuk, P. Royer, P. Samara-Ratna, B. Samuelson, B. A. Sargent, S. Scheithauer, A. Schneider, J. Schreiber, B. Shaughnessy, E. Sheehan, I. Shivaei, G. C. Sloan, L. Tamas, K. Teague, T. Temim, T. Tikkanen, S. Tustain, E. F. van Dishoeck, B. Vandenbussche, M. Weilert, P. Whitehouse and S. Wolff, PASP, 135, 048003 (2023).
